# Right coronary artery perforation extending to the coronary sinus of Valsalva during percutaneous intervention successfully sealed with polytetrafluoroethylene-covered stent: a case report

**DOI:** 10.1186/s13104-017-2867-3

**Published:** 2017-10-30

**Authors:** Sahela Nasrin, Fathima Aaysha Cader, M. Maksumul Haq, Md. Rezaul Karim

**Affiliations:** 1 Department of Cardiology, Ibrahim Cardiac Hospital & Research Institute (ICHRI), Dhaka, Bangladesh; 2 National Institute of Cardiovascular Diseases, Dhaka, Bangladesh

**Keywords:** Coronary artery perforation, Sinus of Valsalva, Covered stent

## Abstract

**Background:**

Right coronary artery perforation extending to the sinus of Valsalva is a rare and potentially fatal complication of percutaneous coronary intervention. There are no definite guidelines on the management strategies for such complications. Treatment modality depends on the patient’s haemodynamic stability and the extent of aortic involvement. Polytetrafluoroethylene-covered stents have emerged as a revolutionary strategy, enabling efficient endovascular repair of the entry port of such dissections, particularly the coronary ostia, and obviating the need for high-risk emergent surgical intervention.

**Case presentation:**

A 60 year old Bangladeshi gentleman underwent a coronary angiogram following a prior inferior ST elevation myocardial infarction (MI), 1 month previously. Coronary angiography done via right radial approach using 5 FR TIG catheter showed diffuse mid RCA disease with maximum 90% stenosis. Angioplasty of the RCA was planned. The RCA was cannulated with a 6-French JR 3.5 guiding catheter (USA). The lesion was crossed by a 0.014 inch guide wire and stented with a 2.75 × 38 mm novolimus-eluting DESyne stent, after predilatation. Immediately after stenting, a Type II perforation was observed in the ostial RCA, which progressed into the right coronary sinus of Valsalva. As the patient was haemodynamically stable with no ischaemia on ECG, we attempted to seal the ostial RCA with bare metal stents. Two successive bare metal stents failed to seal the aorto-coronary dissection. Ultimately, a 3.0 × 19 mm polytetrafluoroethylene-covered stent was deployed to seal the entry port in the ostial RCA, yielding a satisfactory angiographic result with only minimal contrast staining limited to the right sinus of Valsalva. The patient was closely monitored and discharged on dual antiplatelet therapy comprising of aspirin and prasugrel. He remained asymptomatic and with follow up echocardiograms showing no pericardial effusion nor extension of the dissection.

**Conclusions:**

The polytetrafluoroethylene-covered stent provides a safe and effective means of sealing iatrogenic aorto-coronary dissections complicated by Ellis type II or II perforations, thus avoiding emergency surgery. However, as they are associated with increased incidence of stent thrombosis, an efficient and prolonged post-PCI antiplatelet regimen is recommended.

**Electronic supplementary material:**

The online version of this article (10.1186/s13104-017-2867-3) contains supplementary material, which is available to authorized users.

## Background

Iatrogenic right coronary artery (RCA) perforation with extension to the sinus of Valsalva is a rare but potentially life-threatening occurrence during coronary angiography and percutaneous coronary intervention (PCI) [1–6]. Although it may sometimes be difficult to discern between coronary perforation and dissection [7], aorto-coronary dissection is usually diagnosed with angiography showing a persistently localised dye staining within the sinus of Valsalva or extension into the aorta [1, 8]. The gravity of this event hinges on the fact that it could lead to acute occlusion of the relevant coronary artery, in addition to the danger of dissection extending to the ascending aorta and further, if not immediately sealed [3].

This potentially fatal complication is often the result of a coronary artery dissection or perforation providing the entry door for subsequent retrograde progression into the sinus of Valsalva [9]. In the vast majority of cases, it involves percutaneous procedures of the RCA [1–7, 9–22].

Treatment remains challenging, as prompt institution of management is paramount to avoiding fatal outcome. Options include urgent surgical intervention, placement of an intracoronary stent at the origin of the culprit vessel, or conservative treatment [2, 3, 10]. Limited aorto-coronary dissections have been successfully managed by stent implantation with good coverage to the ostium of the perforated/dissected vessel [1–3, 5–7, 15, 16, 19, 22]. In the modern era, polytetrafluoroethylene (PTFE) covered stents have emerged as a revolutionary strategy to seal the entry site, particularly in cases of perforation [6, 7, 10, 23–26]. They are also associated with better long term outcome and prognosis. More extensive involvement of aortic root and/or ascending aorta requires surgical intervention, and is associated with poor prognosis [3, 4, 12, 16]. We report a case of aorto-coronary dissection during PCI, which resulted from extension of a type II perforation of RCA into the Sinus of Valsalva. It was successfully managed with a covered stent, without surgical intervention, resulting in optimal coronary blood flow and diminution of the aortic dissection.

## Case presentation

A 60-year-old normotensive, non-diabetic Bangladeshi male underwent an elective CAG with the indication of unstable angina. He had a history of Inferior ST elevation myocardial infarction (MI), thrombolysed with Streptokinase 1 month earlier at a state tertiary care institute.

Physical examination was unremarkable. Electrocardiography (ECG) showed pathological Q and T inversions in the inferior leads (II, III, aVF) indicative of a prior inferior MI. Trans-thoracic echocardiogram (TTE) revealed a left ventricular ejection fraction (EF) of 50% with inferior wall hypokinesia. All blood investigations were normal including renal parameters. He was on aspirin, clopidogrel, bisoprolol, nitroglycerine, trimetazidine and atorvastatin.

**Fig. 1:**
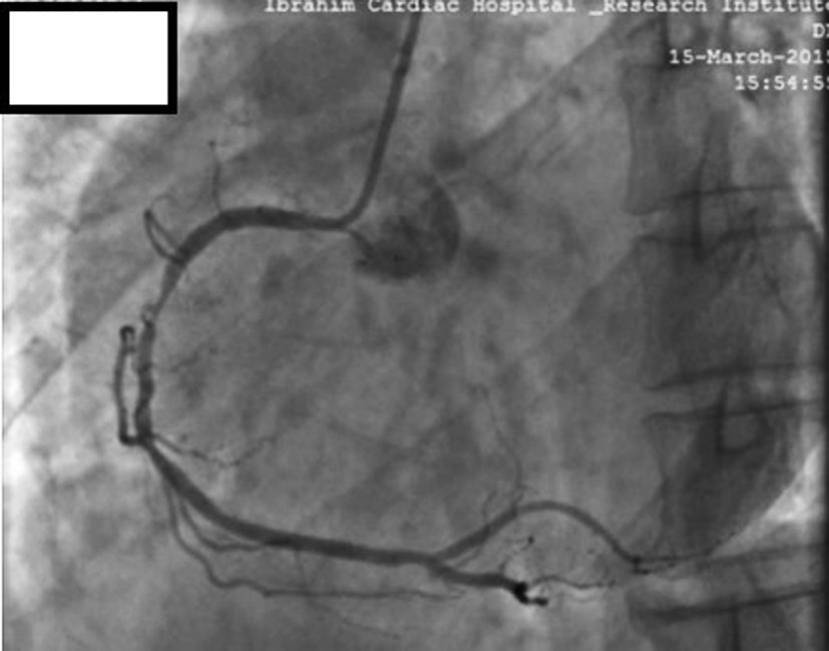
Coronary angiography showing dominant right coronary artery (RCA) and diffuse mid RCA disease with maximum 90% stenosis

He was given a pre-procedural antiplatelet load of 600 mg clopidogrel and CAG was done via right radial approach using 5 FR radial TIG catheter (Terumo). Angiographic procedure was uncomplicated and revealed a dominant RCA and diffuse mid RCA disease with maximum 90% stenosis (Fig. 1). Left anterior descending (LAD) artery and 2nd obtuse marginal (OM2) artery had mild and moderate disease respectively.

**Fig. 2:**
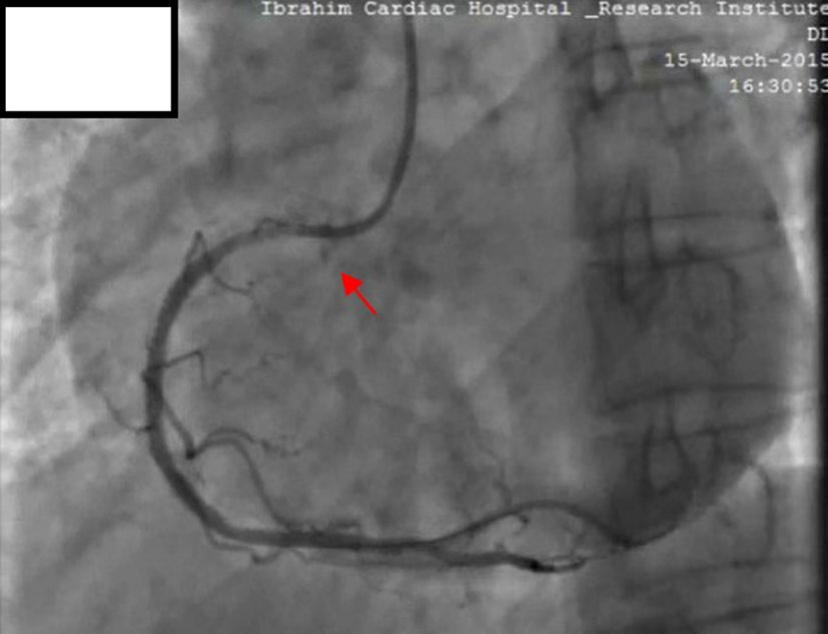
Coronary angiography following stenting of RCA lesion. Stenting was done by 2.75 × 38 mm novolimus-eluting DESyne stent at 16 ATM for 20 s, with restoration of thrombolysis in myocardial infarction (TIMI) III flow. An Ellis type II perforation is noted in the ostial RCA, evidenced by subtle contrast (red arrow)

**Fig. 3:**
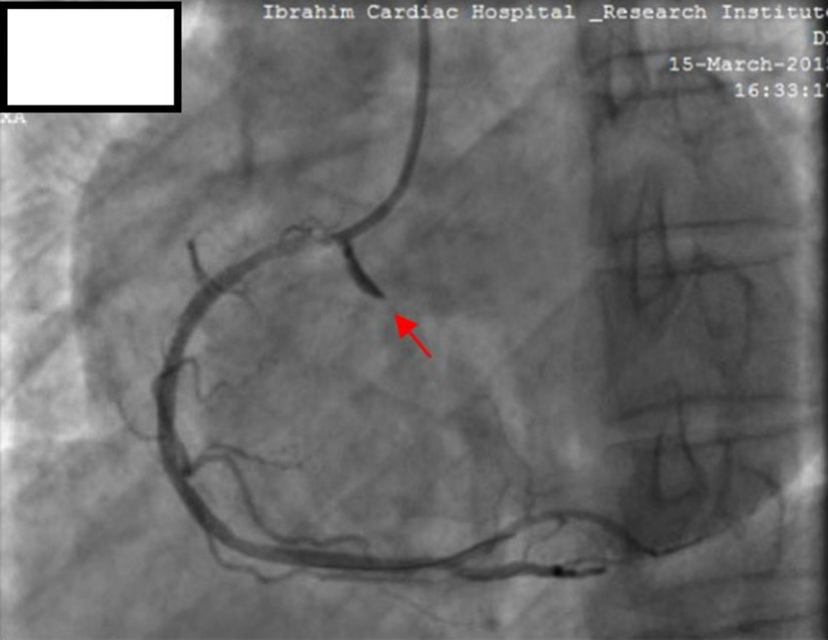
Coronary angiography following the next contrast injection showing extension of RCA perforation into the right coronary sinus of Valsalva (red arrow)

Following CAG, he was selected for angioplasty of the RCA. The RCA was easily cannulated with a soft tipped 6-French JR 3.5 guiding catheter (USA). After the selection of the guiding catheter in the RCA, a 0.014 in. Asahi Sion Blue PTCA guide wire (Asahi Intecc Co., Ltd., Japan) was advanced to cross the lesion. Pre-dilatation was done by 2.5 × 15 mm Sapphire balloon at an inflation pressure of 12 atmospheres (ATM) for 10 s. Stenting was done by 2.75 × 38 mm novolimus-eluting DESyne stent (Elixir medical corporation, Sunyvale, CA) at 16 ATM for 20 s, with restoration of thrombolysis in myocardial infarction (TIMI) III flow. At this point we observed an Ellis type II perforation in the ostial RCA, as evidenced by subtle contrast (Fig. 2; Additional file 1: Video S1). The next contrast injection given very gently showed that the perforation had extended into the right coronary sinus of Valsalva, as evidenced by contrast dye beyond the RCA ostium and retrograde stasis limited to the sinus of Valsalva (Fig. 3; Additional file 2: Video S2). There was no rupture of the balloon. Despite coronary perforation, the patient remained haemodynamically stable, reported no chest discomfort and electrocardiogram showed no sign of new ischemia. Urgent cath lab TTE showed no pericardial effusion or aortic regurgitation (AR). Serum cardiac enzymes and troponin I were within normal range.

**Fig. 4:**
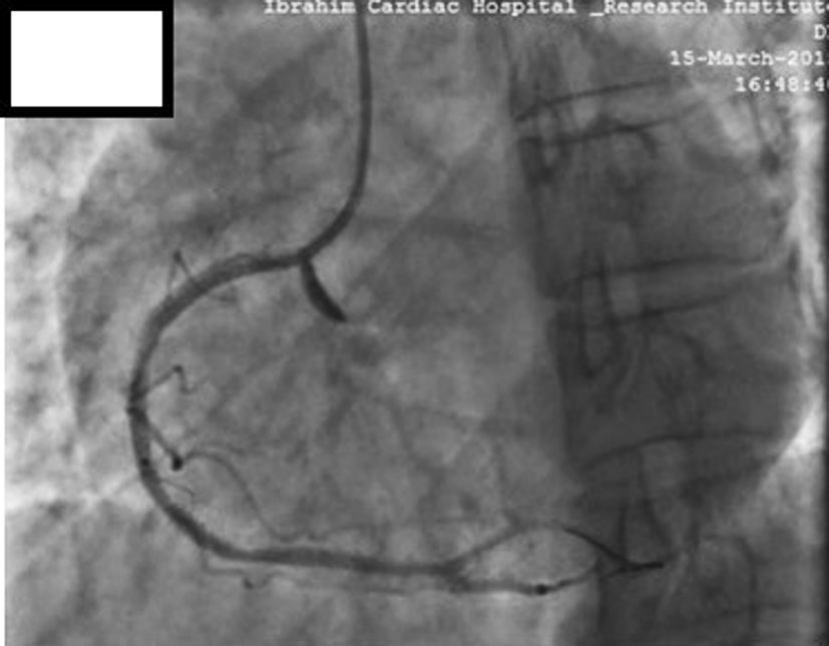
Coronary angiography after implantation of bare metal stent in an attempt to seal the perforation showing persistence of contrast within the sinus of Valsalva. A Genius Magic Eurocor GmbH stent (Germany) Cobalt chromium coronary stent system was deployed in proximal overlap of the previous stent, to obtain adequate coverage of the RCA ostium

**Fig. 5:**
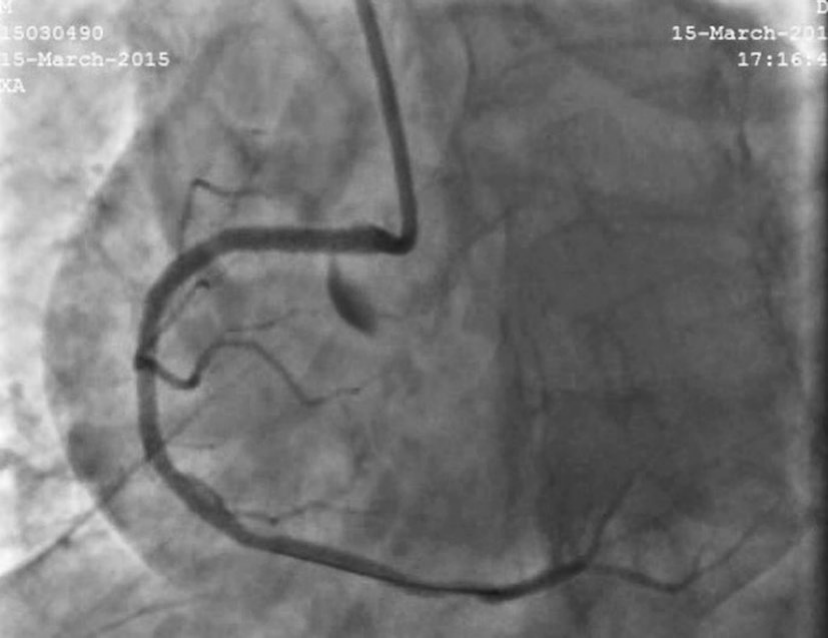
Coronary angiography after implantation of a second bare metal stent in an attempt to seal the perforation showing contrast within the sinus of Valsalva. A 3.0 × 12 mm Avantgarde stent Cobalt chromium coronary stent system was deployed, covering the ostium of RCA, but with no satisfactory result

An attempt to seal the perforation in the ostial RCA was made with a 3.0 × 23 mm bare metal stent (BMS) (Genius Magic Eurocor GmbH stent (Germany) (Cobalt chromium coronary stent system), which was deployed in proximal overlap of the previous stent, to obtain adequate coverage of the RCA ostium (Fig. 4). Despite this, as the dissection persisted, another 3.0 × 12 mm BMS (Avantgarde stent Cobalt chromium coronary stent system) was deployed, covering the ostium of RCA, but with no satisfactory result (Fig. 5).

**Fig. 6:**
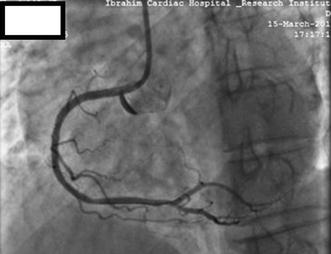
Coronary angiography after implantation of PTFE-covered stent showing minimal contrast staining limited to the right sinus of Valsalva. A 3 × 19 mm Graftmaster RX Coronary Stent Graft System, Abbott Vascular, USA was deployed in proximal overlap to include complete coverage of the ostial RCA with good angiographic result and TIMI Grade III flow

Ultimately, a 3.0 × 19 mm PTFE-covered stent (Graftmaster RX Coronary Stent Graft System, Abbott Vascular, USA) was deployed in proximal overlap to include complete coverage of the ostial RCA, which was considered the entry point of the aortic dissection. The next control injection demonstrated satisfactory sealing of the coronary perforation with only minimal contrast staining limited to the right sinus of Valsalva. The RCA presented a good angiographic result with TIMI Grade III flow (Fig. 6; Additional file 3: Video S3). A final cine run taken 30 min later showed a faintly opacified coronary sinus and no further extension into the aortic root.

As the patient experienced no chest pain or ischaemia, the procedure was concluded and he was monitored in the coronary care unit (CCU), where he remained haemodynamically stable throughout. Follow up TTE done daily showed no signs of pericardial effusion, AR or further progression of the dissection. He had an uneventful post-catheterisation period and was discharged 2 days later on dual antiplatelet therapy (DAPT) of aspirin and prasugrel, in addition to anti-ischaemic medications. The patient was asymptomatic at 2-week and subsequent follow up at 2 months, and TTE showed no pericardial effusion.

## Discussion and conclusions

Despite its infrequent incidence, aorto-coronary dissection involving the sinus of Valsalva is potentially catastrophic, leading to devastating consequences [3–6, 11, 13, 20, 21, 27]. The first case of aortic dissection as a complication of PCI was described by Moles et al. [4]. As in our case, the majority (i.e. > 80%) of such cases have arisen secondary to RCA dissection or perforation [1–7, 9–22, 27], usually the proximal or ostial RCA [8]. This could be due to differences in the histologic structure of the ostia of RCA and left main coronary artery (LMCA) [1, 8, 12, 18]. The periostial and sino tubular junctions of LMCA are formed by more concentrically arranged circular and spiral smooth muscle cells in its intima, with abundant elastic fibres than the RCA, which explains the LMCA’s greater resistance to retrograde dissection) [1, 8, 12, 18].

In the pre-stent era, dissection of sinus of Valsalva was reported secondary to prolonged balloon inflation, associated with rupture of the balloon [3, 22]. With the advent of PCI and stenting, the mechanisms for iatrogenic perforation are being attributed to: aggressive manipulation of rigid wires into subendothelial spaces, when attempting to re-canalise tight lesions [3, 11, 12, 14, 16, 19, 22]; forceful manipulation of guiding catheters that are either wedged [17] or in a non-coaxial position relative to the proximal segment of the coronary artery [2–4, 6, 8, 10–15, 17, 18, 20, 22, 27, 28]; and prolonged balloon inflations [3, 12]. A vigorous manual injections of contrast medium into the subintimal space plays a major role in extending the dissection further, by generating reverse flow proximal to the catheter tip and causing retrograde extension [3, 4, 8, 11, 14, 15, 17–19, 21, 28].

Perhaps the greatest risk for PCI-related dissections is the presence of heavily calcified vessels or chronic total occlusions (CTO) [13, 22], which require more aggressive catheter manipulation for better support, and the usage of stiff wires in vessels whose walls are less resistant to trauma [1, 2, 8, 11, 14–16]. Hydrophilic coated guide wires are associated with increased risk of coronary perforation [7, 21, 26, 29], due to low coefficient of friction and ease of distal migration [29]. Amplatz catheters are more frequently associated with ostial coronary artery dissections [2, 7, 15, 21]. Some reports have also demonstrated the involvement of JR catheters in dissection [1, 2, 7].

A recent MI, as in our patient, poses additional risk, as the inflammatory process of infarcted vessels render them tender, with tendency to dissection [1, 2]. Iatrogenic dissections are more common in the setting of acute MI (0.19%), than for non-acute MI interventions (0.01%) [2]. Cystic medial necrosis [3, 11, 17], smaller RCA size [2], arterial hypertension and age > 60 years are additional risk factors [1].

In our case, mechanical trauma caused by guide catheter manipulation during its withdrawal seems the most likely culprit for causing the port for further dissection: shearing forces of blood flow during systole or diastole may have assisted the propagation of the ostial perforation to involve the sinus of Valsalva [1.4.21]. Even vigorous inspiration by the patient during contrast injection could pose a risk [30] as in our patient. Moreover, he was restless throughout the procedure, repeatedly attempting to observe it, in spite of strong counselling against doing so.

Once the dissection was noted, vigorous contrast injections were avoided and further stenting was done with gentle manipulations of guide catheter and wires. Rapid recognition of the patient’s haemodynamic conditions [20] and urgent assessment by TTE or trans-oesophageal echocardiography (TOE) in the cath lab [14] is paramount to deciding treatment modality, which also depends on the extent of aortic involvement and underlying coronary anatomy [2, 5, 16].

In our case, there was an Ellis type II perforation in the proximal RCA which extended into the ostium and further into the sinus of Valsalva. Ellis et al. defined the angiographic classification of coronary perforations, describing type II perforations as pericardial or myocardial blush without a ≥ 1 mm exit hole [31]. Type I and type II perforations are usually confirmed by retrospective review of the angiogram, and can be easily missed if not looked for [7].

Localised dissections are usually contained below the sino-tubular junctions, by the well-developed supra-valvular ridge, and believed to resolve spontaneously in the first month [1, 3, 8]. However, despite some successful outcomes [8, 13, 20, 32], localised dissections treated conservatively have sometimes rapidly progressed to extensive aortic dissection [3, 18, 22], with some even subsequently requiring surgical intervention [1, 3, 4].

Thus, immediate percutaneous stenting of the perforated ostium and sealing the presumed site of entry door for aorto-coronary dissection, is the preferred modality in haemodynamically stable and localised dissections (with suitable anatomy for stenting) [1, 5, 6, 12, 19, 22]. Bae et al. reported the first case of aorto-coronary dissection successfully treated with PTCA and stenting, without an operation [19].

Previously, bare metal stents were preferred, owing to their delivery ability in the ostium [11, 13, 16]. Some operators have deployed drug-eluting stents (DES) [11, 21]. However, the presence of cells in a standard stent may not fully cover the site of entry of the dissection [6]. Thus, in free perforations causing a large entry port at the coronary ostium, stent grafts or covered stents are more effective [8, 15], as they prevent contact between vessel wall and components of blood, acting as a mechanical barrier [6, 33, 34]. Successful coronary stenting seals off the entry port within minutes of the complication developing, thus preventing further propagation of aortic dissection [1, 3, 6, 8, 21].

Abu-Ful et al. reported the first case in which acute aortic dissection complicating PCI was treated with a covered stent to seal the entry site [6], a strategy that was later adopted by others [7, 10]. The Graftmaster covered stent comprises of an expandable PTFE graft material sandwiched between two coaxially aligned stainless steel stents [6, 33]. They are ideally suited to fully cover and seal entry sites formed by the coronary ostium itself, where standard stents maybe insufficient [6, 33]. However, as they are bulky with low flexibility and high profile, delivery may be difficult, and high-pressure deployment and/or IVUS-guided optimisation has been recommended [13, 24, 33].

PTFE stents are associated with increased incidence of subacute stent thrombosis higher than in standard stents, estimated at 5.7 to 8.6% [23, 33, 34] and can occur later than with conventional stents. Increased incidence of restenosis and target lesion intervention is also reported [7, 33, 34]. These have been attributed to increased thrombogenicity and delayed endothelialisation of these stents [23, 26], thus warranting an extended duration of DAPT of at least 6 months to 1 year [24, 26, 34, 35]. Prasugrel or ticagrelor are suitable antiplatelet agents of choice, due mainly to their lack of intrinsic resistance [23, 35].

Once the dissection is stabilized, careful monitoring with optimum BP control and non-invasive imaging techniques, preferably in a coronary care unit setting is required, to detect progression of the dissection and ensuing complications. This can be accomplished by serial TTE/TOE, CT or MRI scan [3, 11, 12, 16, 18]. TOE/TTE appears to be superior to CT in the follow up of patients [1]. TOE has also been recommended instead of angiography [3]. If extension of the dissection is demonstrated, surgical repair should be considered [12].

This case demonstrates the greater efficacy of covered stents over bare metal stents, in immediate sealing of coronary ostium, thus limiting aorto-coronary dissections localised to the sinus of Valsalva. In addition to minimal adverse effects, covered stents also circumvents the additional risks otherwise posed by the alternative option, which is surgical intervention and are associated with better prognosis [22]. However, appropriate treatment modalities should be reviewed on a case-by-case basis, particularly taking into account the patient’s haemodynamic conditions and coronary lesion morphology.

## Additional files

**Additional file 1: Video S1.** Coronary angiography following stenting of RCA lesion. Stenting was done by 2.75 × 38 mm novolimus-eluting DESyne stent at 16 ATM for 20 s, with restoration of thrombolysis in myocardial infarction (TIMI) III flow. An Ellis type II perforation is noted in the ostial RCA, evidenced by subtle contrast.

**Additional file 2: Video S2.** Coronary angiography following the next contrast injection after Ellis type II perforation. Angiography showing further extension of RCA perforation into the right coronary sinus of Valsalva.

**Additional file 3: Video S3.** Coronary angiography after implantation of PTFE-covered stent. There is significant of reduction contrast staining of the right coronary sinus of Valsalva following covered stent implantation.

## Abbreviations

RCA: right coronary artery
CAG: coronary angiography
PCI: percutaneous coronary intervention
PTFE: polytetrafluoroethylene
MI: myocardial infarction
ECG: electrocardiogram
TTE: tran-thoracic echocardiography
EF: ejection fraction
LAD: left anterior descending
OM: obtuse marginal
ATM: atmospheres
TIMI: thrombolysis in myocardial infarction
BMS: bare metal stent
CCU: coronary care unit
DAPT: dual antiplatelet therapy
LMCA: left main coronary artery
CTO: chronic total occlusion
TOE: trans oesophageal echocardiography
DES: drug eluting stent
